# Precipitation regime change in Western North America: The role of Atmospheric Rivers

**DOI:** 10.1038/s41598-019-46169-w

**Published:** 2019-07-09

**Authors:** Alexander Gershunov, Tamara Shulgina, Rachel E. S. Clemesha, Kristen Guirguis, David W. Pierce, Michael D. Dettinger, David A. Lavers, Daniel R. Cayan, Suraj D. Polade, Julie Kalansky, F. Martin Ralph

**Affiliations:** 1 Center for Western Weather and Water Extremes (CW3E), Scripps Institution of Oceanography, University of California, San Diego, USA; 2 Climate, Atmospheric Science and Physical Oceanography (CASPO), Scripps Institution of Oceanography, University of California, San Diego, USA; 3 United States Geologic Survey, Carson City, Nevada USA; 4 European Centre for Medium-Range Weather Forecasts (ECMWF), Reading, UK; 5 Finnish Meteorological Institute, Helsinki, Finland

**Keywords:** Atmospheric dynamics, Projection and prediction

## Abstract

Daily precipitation in California has been projected to become less frequent even as precipitation extremes intensify, leading to uncertainty in the overall response to climate warming. Precipitation extremes are historically associated with Atmospheric Rivers (ARs). Sixteen global climate models are evaluated for realism in modeled historical AR behavior and contribution of the resulting daily precipitation to annual total precipitation over Western North America. The five most realistic models display consistent changes in future AR behavior, constraining the spread of the full ensemble. They, moreover, project increasing year-to-year variability of total annual precipitation, particularly over California, where change in total annual precipitation is not projected with confidence. Focusing on three representative river basins along the West Coast, we show that, while the decrease in precipitation frequency is mostly due to non-AR events, the increase in heavy and extreme precipitation is almost entirely due to ARs. This research demonstrates that examining meteorological causes of precipitation regime change can lead to better and more nuanced understanding of climate projections. It highlights the critical role of future changes in ARs to Western water resources, especially over California.

## Introduction

### Atmospheric rivers and west coast precipitation volatility

Coastal western North America (West Coast) receives much of its annual precipitation in the form of orographic heavy rain and snow produced by atmospheric rivers (ARs)^1–3^. These “rivers in the sky” deliver intense pulses of water vapor onshore and largely drive the hydroclimate of this region^3–5^. This is particularly true in California, where against the backdrop of recent dryness and persistently mounting anomalous warmth, the notorious volatility of the state’s water resources^3^ has been on display. Only four wet years have occurred so far in the 21^st^ century (water years 2005, 2011, 2017 and 2019). The most recent period included five years of historic drought (2012–2016) with the first three years constituting an exceptionally dry period (water years 2012–2014) in the instrumental record spanning over 120 years^6^. Water year 2014, which tied for the driest year on record, was followed by 2015 – the year of unprecedented warmth and snow drought^7^ (5% of normal snow accumulation in the Sierra Nevada^8^). These dry years were followed by the wettest water year on record for much of California^9–11^ – 2017 – a wet season marked by widespread flooding^12^ and AR activity unprecedented in seven decades of record^5^. This deluge was then followed by a dry water year 2018, especially in Southern California, parts of which received less than 1/3 of normal precipitation. Yet, even 2018 was punctuated with anomalously warm flood-producing storms^13^. Currently, a long series of alternating cold frontal and warm AR storms is resulting in a very wet water year 2019 in California featuring AR-driven flooding^14^.

This volatility is associated with California’s Mediterranean climate, which generates annual precipitation in a narrow window of opportunity during the cool season^3^. Recent extreme hydroclimatic variation over the region was notably marked with unprecedented warmth. The heightened water resource volatility and associated impacts exemplify expectations from a warming climate in Mediterranean California, where precipitation will be delivered with progressively declining frequency but increasing intensity^15,16^. In climate-change projections, shrinking numbers of storms producing the region’s annual total precipitation result in increasing year-to-year variability of water resources^15,17^. Specifically, as the Hadley Cell widens^18,19^, California, along with the other Mediterranean climate regions, becomes more subtropical^16,18,20^. Frequency of precipitation declines mainly in fall and spring^21^, which results in decreased shoulder-season precipitation^17,22^, while heavy precipitation becomes preferentially more extreme in winter^16^ – the AR season in California^5^. The meteorological mechanisms of this regional precipitation regime change, however, have not been quantified and are currently inadequately understood.

Atmospheric rivers along the West Coast can produce in excess of 50% of total annual precipitation^5^ and are historically associated with extreme precipitation and flooding^3,23^. They can also penetrate far inland along preferred topographic corridors^24–26^. Consequently, the majority of western floods are orchestrated by ARs land-falling upon the West Coast^22^ and so are flood insurance claims^27^. Thermodynamic enhancement of vertically **i**ntegrated horizontal water **v**apor **t**ransport (IVT) in a warmer future^28^ will lead to wetter, wider and longer ARs globally^29^, exacerbating their impacts. For the West Coast, evidence of increasing IVT^30^ as well as of AR landfalling activity associated with observed long-term Pacific sea surface temperature (SST) warming has been reported with impacts on precipitation over the West, although this trend has not heretofore clearly emerged from the envelope of natural variability^5^. Increasing IVT and associated AR activity have been clearly projected over the West Coast for later in the century^31–35^. Although some of these model studies linked changes in AR activity to precipitation accumulations, particularly from heavy precipitation events assessed at the coarse GCM resolution^34,35^, the existing body of work on IVT and atmospheric rivers in climate model projections has not so far fully considered impacts on precipitation regimes, especially at the scales relevant for driving regional water resources. Since much of the precipitation produced by ARs is orographic, such information is essential at a fine enough scale to resolve spatial precipitation patterns influenced by the region’s complex topography, which importantly includes its Coastal Ranges as part of the greater North American Cordillera.

We here explore how landfalling ARs are projected to change along the West Coast and, using statistically downscaled bias-corrected precipitation^36^, how these changes play into the precipitation regime change projected for this region^15–17^ by a set of 16 GCMs under the “business-as-usual” emissions scenario. Validating these models with respect to their realism in simulating historical regional AR activity and its contribution to Western precipitation proves an essential exercise and turns out to constrain projection uncertainty. Focusing on three flood-prone coastal river basins — Chehalis in Washington State, Russian in Northern California, and Santa Ana in Southern California — we examine the changing contribution of ARs to daily precipitation for different intensity categories. Regionalizing these detailed local results to clarify impacts on the precipitation regime over the entire West, we highlight the meteorological causes and nuances of the projected trends in the regional hydroclimate, which are particularly noteworthy over California.

### AR detection and GCM validation – a summary

An automated AR detection scheme^5^ (ARDT) was applied daily to an ensemble of 16 Global Climate Models (GCMs, Table S1) over the historical period (1950–2005) and future (2006–2100) projected under Representative Concentration Pathway 8.5 (RCP8.5) - essentially the “business-as-usual” scenario from Phase 5 of the Coupled Model Intercomparison Project (CMIP5). We have evaluated the GCMs with respect to their ability to realistically reproduce key statistical features of AR land-falling activity along the West Coast. The distinct seasonal cycle of landfall frequencies and intensities as well as the contribution of ARs to total annual precipitation were evaluated, and historical GCM climatologies of these variables were compared to observations (Fig. S1). *Observed* AR activity is derived from a reanalysis (R1)^37^ product and referred to here as the SIO R1 catalog^5^ and precipitation is from gridded station data^38^. GCM precipitation data were downscaled daily using a state-of-the-art statistical downscaling method^36^. The five most realistic GCMs, hereinafter referred to as “Real-5”, were identified and highlighted in Table S1 and in the analyses that follow. Details of GCM data and validation are discussed in Data and Methods.

Projected changes are mainly quantified as differences between the second half of the 20th century (1951–2000 water years: *historical period*) and that of the 21st (2051–2100: *future period*), although changes occurring by and in the *current* (2001–2050) half-century and throughout the 150 years of the CMIP5 record are also assessed. The years referred to here encompass the AR year along the West Coast (from July 1 to June 30). These are numbered consistently with Water Years, i.e. an AR landfalling in November of year *n* contributes to AR Year *n* + 1.

### Historical and projected AR activity

**Figure 1:**
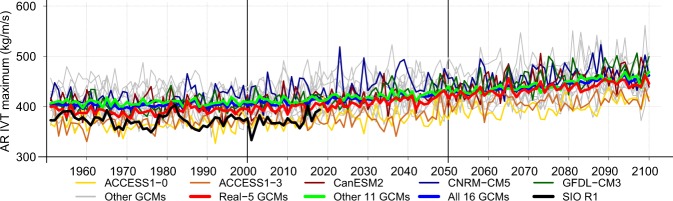
Annual average maximum IVT for AR events landfalling upon the West Coast [20–60°N] in historical (1951–2005, left) and projected (2006–2100, right) epochs. Real-5 GCMs are plotted in thin colored lines, while other GCMs are outlined in gray. Thick curves represent the ensemble averages of the Real-5 GCMs (red), the other 11 GCMs (green), and the full ensemble of 16 GCMs (blue). The thick black curve shows the observed (SIO-R1) variability.

Models simulate a broad range of AR land-falling activities (Figs 1, S2 and Table S2). Some GCMs are unrealistically dry, but most are wet, some severely overestimating AR activity, i.e. event maximum intensity (Fig. 1), frequency (Fig. S2a) and duration (Fig. S2b). The Real-5 GCMs are well in line with observed AR activity. No consistent historical trends are clearly evident in the models’ AR intensity, frequency and duration time series overall (Table S3). The observed AR activity (represented by the SIO R1 catalog^5^), however, has declined over the West Coast, significantly with respect to duration, during the historical period. This is likely due to natural decadal variability and may explain the lack of increasing trends in observed intense precipitation in the West^39^. The models’ internal variability, which largely determines trends during the historical period, is obviously not expected to be in phase with that observed and can result in trends of different sign in different model runs^40^. The observed negative trend is consistent with 6 of the 16 GCM historical realizations (Table S3). During the current half-century (2001–2050), the forced response begins to dominate and AR activity in GCMs begins to clearly trend up. When the GCMs are averaged annually, which filters natural variability, the ensemble average trends emerge as significant in all variables in the Real-5, Other, and Full Ensembles. AR activity ramps up and continues to rise until the end of the century significantly in all GCMs. For AR landfalls along the California coast (rather than the entire West Coast; Fig. S3), trend significance (Table S4) is limited by the larger natural variability associated with the shorter coastline and consequently a smaller sample of ARs landfalling there. Nevertheless, upward trends begin to clearly emerge as significant during the current half-century and continue to intensify in coming decades.

The exercise of model validation with respect to the realism of simulated AR activity (Fig. S1) allowed us to constrain the range of GCM projections considered. For example, Table S2 shows that, while projected increase in West Coast AR event frequency ranges from 4–35% across all models, the range for the Real-5 models narrows to 19–26% (a statistically significant result, see Section 5e). Therefore, unless otherwise stated, the results presented below concentrate on the most realistic models by averaging across the Real-5 models highlighted in Table S2.

Pronounced seasonality organizes AR landfalls with Pacific Northwest (~45–60°N) activity peaking in the late summer/early fall, while California AR activity peaks in winter^5,25,30^. Global warming manifests as a general bolstering of this climatological AR seasonality, making landfalls more frequent and intense at the peak of the AR season as well as broadening the season, particularly forward in time, i.e. deeper into winter (Fig. S4), even as the frequency of precipitation from other more common storms (mainly frontal cyclones) decreases, particularly in the shoulder seasons and in California^21^. Along with enhanced frequency and intensity of AR landfalls, the duration of ARs is also projected to increase (Fig. S4). This is not surprising given the increase in AR intensity combined with use of fixed IVT (>250 kg/m/s) and integrated water vapor (IWV) thresholds to define ARs^29^.

### The role of ARs in projected precipitation regime change

**Figure 2:**
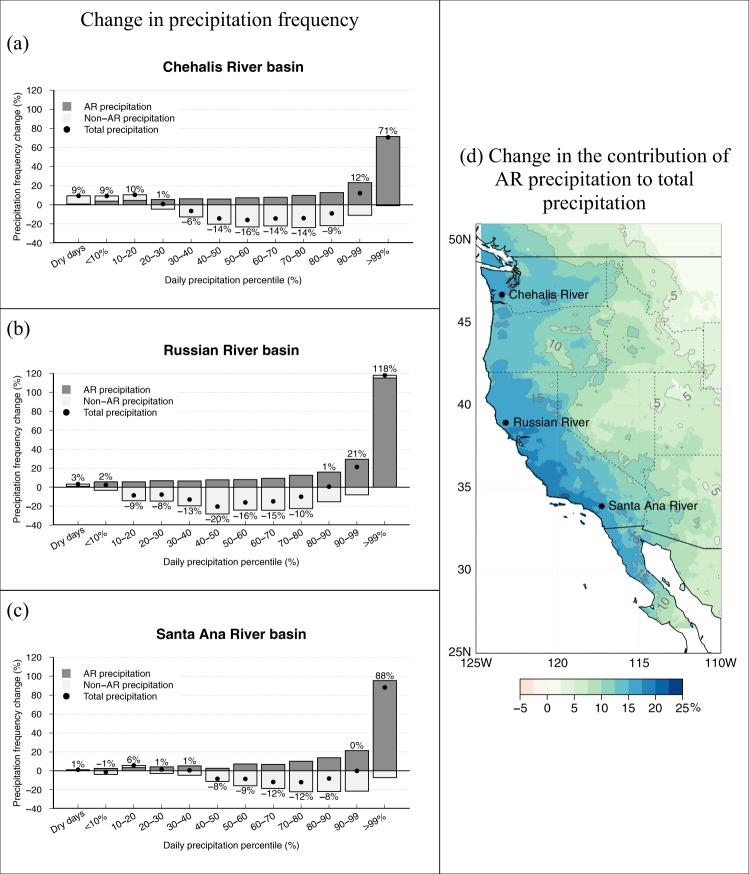
Future changes in daily precipitation frequency binned by percentile ranges of daily intensity (% of historical climatology). Results represent ensemble averages for the Real-5 LOCA-downscaled GCMs for the Chehalis, Russian and Santa Ana river basins (**a**–**c**, respectively). Changes in total precipitation are denoted by dots and associated values; AR-related precipitation (for each AR day and the following day) – dark grey bars; and non-AR precipitation – light grey bars. Panel (d) illustrates Real-5 ensemble average change in the contribution of AR-related precipitation to total precipitation (in % of historical contribution see Fig. S7d,f).

We now consider the contribution of atmospheric rivers to projected changes in precipitation frequency as a function of daily precipitation intensity. The Chehalis, Russian, and Santa Ana river basins were chosen to represent projected conditions in the northern, middle and southern reaches of our coastal domain, where we have classified daily precipitation into intensity bins ranging from dry days to drizzle, to low, medium and heavy intensity bins defined by deciles of the historical daily precipitation probability distribution, to extreme precipitation above the 99^th^ %-ile of all wet days. Daily precipitation thus binned was further classified as AR- and non-AR-related in both the historical and future periods using the ARDT results. The projected changes of precipitation frequency in each bin are displayed in percent of historical frequencies in Fig. 2(a–c); dots. These changes are distinguished into contributions from AR- and non-AR-related precipitation sources (dark grey and light grey boxes, respectively).

In the Chehalis River basin (Fig. 2a), the projected increase in the frequency of dry days (<0.1 mm), drizzle (0.1 mm <=p < 10th %-ile) and low-intensity precipitation (between the 10th and 20th %-iles) is mostly explained by changes in the frequency of non-AR events. The projected decrease in the frequency of medium-intensity precipitation (between 30^th^ and 90^th^ %-iles) is mostly accounted for by strong declines in non-AR events, while ARs are projected to partially counteract this decrease, i.e. contribute more medium intensity precipitation. As far as heavy (between the 90^th^ and 99th %-iles) and extreme (>99^th^ %-ile) precipitation, frequency of days in these categories increases due to ARs. In fact, AR contributions increase in all precipitation bins, with the increases becoming greater at higher intensities, while non-AR precipitation contribution is projected to become more frequent in the drizzle and other low-intensity precipitation categories, and decreasing at higher intensities.

Further south in the Russian (and Santa Ana) River basin, the contribution of ARs also progressively increases in all precipitation intensity categories up to extreme. In the Russian River basin (Fig. 2b), an overall precipitation frequency decrease is projected especially for medium-intensity precipitation (up to 20% decrease relative to the historical period), which results from weakening contribution by non-AR events. ARs in Northern California - a region of exceptionally strong AR activity^5^ - are projected to greatly increase the frequency of heavy and extreme precipitation events. Extreme precipitation in the Russian River basin becomes more than twice as likely in the second half of the 21^st^ century compared to the second half of the 20^th^. This projected change is due essentially to ARs.

Over the Santa Ana River basin (Fig. 2c) in coastal Southern California, projections also display a notable increase in extreme precipitation frequency that is entirely due to ARs, while non-AR events marginally counteract this trend. Projected heavy precipitation frequency does not change here because the increase in the AR contribution to this category is entirely counteracted by a decrease from non-AR storms. As is the case farther north, medium-intensity event frequencies, on the whole, decline due to decidedly less frequent non-AR events, even while ARs are increasing their contribution to these precipitation intensity categories.

Figure 2d shows that later in the century, ARs will become increasingly important to the total annual precipitation budget throughout the western U.S., but particularly along the California coast. The contribution of AR activity to total precipitation in the future increases by about 15% in the Pacific Northwest and 20% in coastal California. The largest increases (>20%) are projected for the coastal ranges of Southern California and Northern Baja California, particularly for the Transverse Ranges. The pattern of increased contribution of AR-related precipitation to total annual precipitation penetrates inland along preferred topographic corridors, although the changes diminish precipitously with distance from the coast over most of the domain, particularly in the lee of the Sierra Nevada, which provide a significant barrier to the moisture transport. This pattern of increasing contribution of extremes to total precipitation, which we here explain by increasing AR activity, has been assessed over California by previous studies^30,41^.

These results provide insight and nuance into earlier classifications of precipitation frequency changes by intensity categories for California^16,41–43^. Generally speaking, the probability density function of daily precipitation is projected to favor heavy and extreme events due to reinforced AR activity in a warmer climate, while the frequency of medium-low-intensity precipitation dwindles as the wintertime circulation over the north-eastern Pacific generally becomes more anticyclonic^44,45^, the storm track migrates poleward^46^, and mid-latitude cyclones respond by generally diminishing in frequency^44,46^. Other results indicate a strengthening and SE-ward extension of the NE Pacific jet stream in the historical jet exit region west of California^46,47^. This is consistent with a stronger and more SE-ward extensive Aleutian Low, which, in combination with a more poleward-extensive Subtropical High, promotes stronger vapor transport into California^16^ bolstering AR activity there but displacing cold-frontal convection northward. While examination of these somewhat competing dynamical mechanisms in the Real-5 GCMs is beyond the scope of the current work, we reiterate that the largest decrease in precipitation frequency occurs in the shoulder seasons^21^, while the largest increase in extreme precipitation should occur at the peak of the AR season (Fig. S4), which in California is winter.

**Figure 3:**
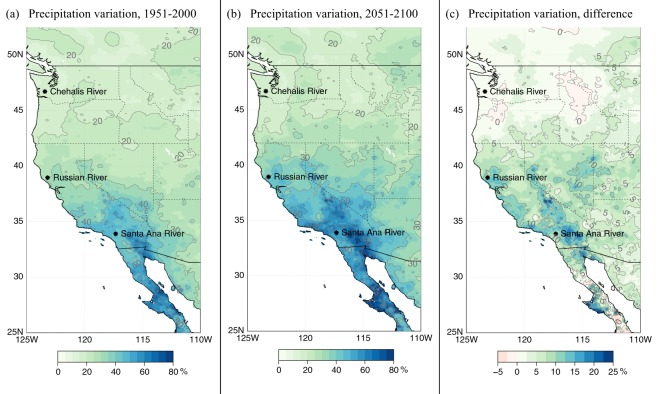
Coefficient of variation (i.e. variance normalized by the mean) of de-trended* annual total precipitation during historical (**a**) and future (**b**) time periods in the Real-5 LOCA-downscaled GCM ensemble average, and the difference (**c**). *150-year polynomial trend was previously removed from the precipitation data (see Section 5f).

**Figure 4:**
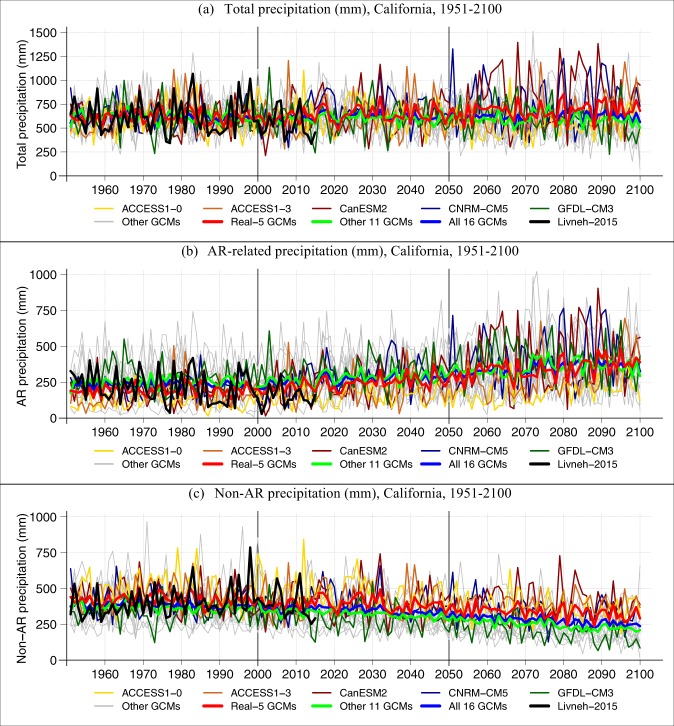
Annual total (**a**), AR-related (**b**) and non-AR related (**c**) LOCA-downscaled precipitation spatially averaged over **California** during historical (1951–2005, left) and projected (2007–2100, right) time periods. Results from the Real-5 GCMs are plotted in thin colored lines, while the Other GCMs are outlined in gray. Thick curves represent the ensemble averages of the Real-5 GCMs (red), the Other 11 GCMs (green) and the full ensemble of 16 GCMs (blue). Thick black curve delineates the annual total (**a**), AR-related (**b**) and non-AR (**c**) precipitation, which is based on observed precipitation data. Trends and their significance are quantified in Table S5.

In summary, in the West, and particularly along the West Coast, AR contributions to total precipitation are increasing (Fig. 2d). The more intense AR-related precipitation drives up average precipitation intensity (Fig. S5), particularly on the WSW- and South-facing slopes of coastal topography, while all precipitation frequency is decreasing (Fig. S6, top row) and the frequency of AR-related precipitation contributions is increasing (Fig. S6, bottom row). In other words, ARs are responsible for the projected increase in total precipitation, specifically over California (Fig. S7). This is in spite of decreasing precipitation frequency there (Fig. S6, top row), which results in greater interannual precipitation volatility (Fig. 3) as more of the annual total is accumulated from a decreasing sample of intensifying, and increasingly AR-related, precipitation events. These results clarify the meteorological mechanisms behind previous results on projected increasing precipitation volatility in California^15–17,48^. Interestingly, the Real-5 GCMs tend to project a wetter future over California (Figs S6 and S7), and although this is significant and noteworthy given that the full ensemble of 16 GCMs is vague on the sign of the overall trend (Fig. 4a, Table S5), this result is likely to have arisen due to random sampling of GCMs (see Section 5e). Also noteworthy is the greater decrease in non-AR-related precipitation projected for California in the *Other* models compared to that in the Real-5 (Fig. 4c, Table S5). The stronger increasing trends in AR-related precipitation and a weaker decreasing trend in non-AR-related precipitation combine to increase the total precipitation in California, according to the Real-5 GCMs as compared to the rest of the full ensemble of 16 GCMs examined here (Table S5). Again, though, we cannot be confident in this significant trend in total annual precipitation given the bootstrapped likelihood of similar trends due to random sampling of GCMs.

The same mechanisms operate over a larger west-coastal domain (Fig. S8), but they do not exert as much impact on precipitation volatility in the Northwest (Fig. 3). Precipitation is much more frequent there and major topography is not oriented ideally to enhance AR-related precipitation intensity (Fig. S5, i.e. NNE-SSW-oriented Cascades compared to NNW-SSE-oriented Sierra Nevada). This results in little change in annual total precipitation (Fig. S7) as well as its interannual variability over the Pacific Northwest (Fig. 3). As nature does not respect political boundaries, Northern Baja California’s border region displays anthropogenic signals similar to those of Southern California, but projections indicate drying further to the south (Fig. S7), where precipitation frequency decreases more decisively (Fig. S6) but precipitation intensity does not follow suit (Fig. S5). In the rain-shadow of the Sierra Nevada, the Great Basin is projected to receive more total precipitation (Fig. S7c), stemming from more frequent precipitation (Fig. S6c) and greater intensity (Fig. S5), although these changes may be less related to AR activity (Fig. S6f) than they are to changes in the Southwest monsoon^49^, which is beyond the scope of the present work.

### Summary, impacts and research needs

Annual precipitation is already more variable year-to-year in California than anywhere else in the United States^31,50^. Climate projections show this variability increasing^15–17,48^, which is to be expected given the increasingly flashy precipitation regime, i.e. smaller sample size of storms providing the annual total precipitation resulting in greater sampling variability^15^. Here we show that precipitation delivered by land-falling ARs along the West Coast will be enhanced by climate change, resulting in more frequent and stronger precipitation extremes even as the overall frequency of precipitation decreases due to fewer non-AR storms. California’s Mediterranean climate setting and the geographical orientation of its mountain ranges amplify this change, increasing precipitation volatility. With its optimal orientation perpendicular to the prevailing direction of vapor transport, the Sierra Nevada squeezes moisture out of ARs on the windward side and leaves a pronounced rain shadow to the east. The same can be largely said about the Coastal Ranges of California and Baja California Norte. Thus, precipitation enhancement is strongest via ARs in California, where already infrequent precipitation is declining due to dwindling non-AR storms, ARs are strengthening and becoming even bigger contributors to the annual total, and topography is ideally aligned to extract increasingly heavy precipitation from strengthening ARs.

Elsewhere along the West Coast, decreased precipitation frequency and enhanced ARs are also projected. However, these changes superimposed on the much wetter and steadier hydroclimate of the Pacific Northwest lead to less dramatic changes in both total precipitation and its interannual volatility. Severe interannual variability marking recent hydroclimatic evolution in California, with predominant drought conditions punctuated by extreme wet years, exemplifies the expected changes in volatility even while obscuring any emerging long-term mean trends. However, the CMIP5 projections scrutinized here indicate that anthropogenic effects are already influencing ARs in our region despite the severe year-to-year volatility. Leaning on many previous studies^28,31–35^, we attribute these trends mainly to thermodynamic moistening of ARs, which results directly in more orographic precipitation. Dynamical circulation changes are also possible^16,33,34,46,47^ and, although we notice hints at changing AR dynamics in the projections, they are vague and highly model-dependent.

Our most robust conclusions with respect to total annual precipitation point at increasing year-to-year precipitation variability due to decreasing frequency of storms making up California’s annual total precipitation and greater contribution of AR-related extreme events. The Real-5 GCMs, however, also project increasing mean annual precipitation, in contrast to the full model ensemble, which is unclear on the sign of the future annual precipitation change. This result is seemingly in agreement with another recent study^51^, where a different subset of GCMs was selected based on criteria related to El Niño teleconnection realism. The projected wetter conditions by the Real-5 GCMs, however, are likely to be due simply to random sampling variability (i.e. sampling of GCMs from the full ensemble) as suggested by our bootstrap resampling test (see Section 5e). Given the leading role of ARs in driving the region’s annual precipitation, it is reasonable to consider ability to realistically simulate AR behavior as prerequisite to realistic projections of future regional hydroclimate. This, however, is only part of the story. In the Real-5 GCMs, the stronger increasing trends in AR-related precipitation combine with weaker decreasing trends in non-AR-related precipitation to produce an overall increase in total California precipitation as compared to the rest of the full ensemble of 16 GCMs examined here. In any case, notwithstanding the challenges stemming from a more volatile precipitation regime, California can take solace in the fact that it is not projected to dry as are the other Mediterranean climate regions around the world^16^.

These results highlight the importance of validating models with respect to their realism in simulating the salient regional weather and climate features that are central to the study at hand. In this study, the validation exercise yielded a significant reduction in the range of projected AR behavior, resulting in a more constrained precipitation regime projection. Past studies have not been successful in reducing projection uncertainty^41,52^ as they culled GCMs based on their abilities to simulate several general features of regional climates, while more targeted recent studies conclude that weighting all models equally is suboptimal^53–55^. Our results highlight the importance of focusing GCM validation on the mechanistic response that matters to the study at hand. Our results also highlight the outstanding need to understand why many models have trouble realistically simulating the observed climatology of AR behavior as well as AR contribution to total precipitation.

Understanding the driving mechanisms of hydroclimatic change leads to a more nuanced perspective on West Coast impacts of climate warming. The emerging trends will challenge water resource and risk management. The challenges faced by West Coast reservoir managers in balancing the mandates for water storage and flood control will escalate with an increasingly volatile precipitation regime. Exacerbating these mounting challenges, ARs tend to be warm storms, which in a warmer world will yield progressively higher rain/snow ratios, resulting in an additional boost to runoff in winter and added threat to an already dwindling snowpack^56^ from rain on snow events^57,58^. The climate warming could thus operate via an additional mechanism of warmer and stronger ARs to enhance snowpack erosion from lower elevations progressively upwards. Enhanced runoff generated by boosted and warmer rainfall extremes in a flashier precipitation regime will likely also adversely impact groundwater recharge and storage^59^. The impacts of hazards such as floods^23,27^, debris flows^60^, and coastal water quality^61^ will be exacerbated by the changing precipitation regime alongside the more regionally extensive impacts of drought^62^.

As ARs and their associated precipitation become more extreme, the need for improved predictions of AR activity on timescales ranging from days to seasons will grow in order to better manage the consequences of the changing climate. This is given limited current predictability of AR landfalls^63^ and their important role in driving the water resources of the West as well as the numerous other societal impacts associated with AR landfalls or lack thereof. Beyond California and western North America, ARs can be expected to play increasingly important roles in all AR-prone regions of the world: mainly west- and equatorward-facing coasts of subtropical, mid- and high-latitude land masses from Greenland to Antarctica^29,64–66^. In the warming climate, thermodynamics will ensure the increasing role of ARs in the hydroclimates of all these regions with dynamical drivers of change possibly adding important regional detail to this evolving global picture.

## Data and Methods

### Global climate model (GCM) data

The study considers 16 GCMs (Table S1) from the *Phase 5 of Coupled Model Intercomparison Project* (CMIP5^67^), for which the daily data was available for computing the vertically integrated horizontal vapor transport (IVT) – the key variable for defining and detecting atmospheric rivers (ARs). For the projected period, we focus on the Representative Concentration Pathway 8.5^67^ (RCP8.5) - essentially the “business-as-usual” scenario from CMIP5. The daily simulations of historical (1950–2005) and projected (2006–2100) specific humidity, zonal and meridional wind components at four standard pressure levels spanning 1000–500 millibars (1000, 850, 700 and 500 mb) were used to estimate IVT [1] and detect ARs landfalling along the west coast of North America (20–60°N).

1 $$IVT=\sqrt{{(\frac{1}{g}{\int }_{1000}^{500}qudp)}^{2}+{(\frac{1}{g}{\int }_{1000}^{500}qvdp)}^{2}},$$

where *q* is specific humidity, *u* is zonal wind, *v* is meridional wind, *p* is pressure, and *g* is acceleration due to gravity.

Twelve of the sixteen GCMs did not consistently report atmospheric data extrapolated to 1000 mb during synoptic low-pressure conditions. In these models, specific humidity and wind vectors near the surface (10 m) were substituted for 1000 mb-level data. These GCMs are marked by an asterisk (*) in Table S1, which provides information on each model: name, origin and spatial resolution. Landfalling ARs were detected at the native resolution of each GCM and the catalogs were interpolated spatially to a common grid (2.5° × 2.5°) and temporally to a standard (Gregorian) calendar. Only the first integrations from the ensembles associated with each GCM were considered.

### AR detection algorithm

The methodology chosen to detect landfalling ARs is that of Gershunov *et al*.^5^ (SIO-R1), which has been developed at the Scripps Institution of Oceanography (SIO) and applied to the National Center for Environmental Prediction and National Center for Atmospheric Research (NCEP/NCAR) reanalysis^37^ (R1) and validated with independent observed precipitation data^5,38^. The SIO-R1 catalog is recognized for its relevance for West Coast precipitation studies and it compares favorably with other available AR catalogs^68,69^. Specifically, this 2.5° × 2.5° R1 catalog is in good agreement with those based on more finely-resolved reanalyses^68^ and provides a longer historical record for GCM validation (1948-present) than most other reanalysis products. The SIO-R1 catalog with adjustment for temporal resolution consistency with daily GCMs data is used as the historical “observational” data for GCM validation.

For consistency with GCM data, we adjusted the 6-hourly AR detection methodology of Gershunov *et al*.^5^ to daily resolution, as follows:

- At each daily time step, spatial patterns of contiguous grid cells with IVT in excess of 250 kg/m/s and IWV in excess of 15 mm are identified.
- Those patterns that cross the coastline and are at least 1500 km long in their entirety (including inland penetration) are retained.
- The grid cell corresponding to the IVT maximum along the coast is considered the “central land-falling location”.
- The central land-falling location of a continuous AR event may not move more than 1000 km (8.9° north or south) from one day to the next. The number of grid cells depends on the GCM/R1 spatial resolution.
- Two AR events making landfall in the same coastal vicinity are considered distinct if they are separated by at least 24 hours.

Daily catalogs of AR landfalling activity were thus created for R1 and each of the 16 GCMs.

### Fine-resolution precipitation data

Daily precipitation data on a 6 × 6 km grid^38^ is used to assess observed AR-related precipitation. Specifically, precipitation associated with ARs is defined as that occurring during AR landfalls and on the subsequent day in regions demarcated by the AR footprint, defined as in Gershunov *et al*.^5^. Localized Constructed Analog statistical downscaling^36^, trained on the Livneh *et al*.^38^ data, was used for the same purpose in the 16 GCMs historical simulations and projections. Importantly, LOCA downscaling includes bias correction^70^.

### GCM validation

The ability of GCMs to accurately represent the historical AR climatology was quantified by comparison against SIO-R1. We validate the GCMs with respect to two metrics. First, the AR landfalling climatology patterns along the West Coast (20–60°N) over 50 years (from July 1950 through June 2000) are assessed via the average frequency of AR days detected by month and landfalling latitude (see Gershunov *et al*.^5^ for calculation details). Second, the climatological contribution of ARs to total annual precipitation (see Gershunov *et al*.^5^ for calculation details). The agreement between GCM- and observed AR climatology patterns was quantified using spatial correlation and root mean square error (RMSE) and summarized in Fig. S1.

The models with the most realistic climatologies with respect to AR day frequency and AR precipitation contribution to the annual total are those with the highest spatial correlation and lowest RMSE (lower right corner of Fig. S1, panels a and b, respectively). We highlight the most realistic five models (Real-5) in Table S1. CNRM-CM5 produces an AR climatology similar to the SIO-R1 reference dataset. These are: CNRM-CM5, CanESM2, ACCESS1-0, ACCESS1-3 and GFDL-CM3. CanESM2, ACCESS1-0 and ACCESS1-3 underestimate AR day frequency, while GFDL-CM3 overestimates AR day frequency during the historical period (see Table S1 in the Supplementary materials). The Real-5 ensemble average AR climatology shows the best agreement (red asterisks on Fig. S1a,b) with the observed climatology. It is noteworthy that the Real-5 GCMs were also independently selected for California Water resources planning^71^ based on key hydroclimate metrics for California, such as those reflecting the Mediterranean climate regime and El Niño - Southern Oscillation (ENSO) teleconnections.

Since we are mindful of results highlighting uncertainty due to internal model variability^40,72^, it is important to note that we used one model realization from each GCM, i.e. the first realization (r1i1p1) from each GCM ensemble. These were the only integrations from each GCM that were entirely downscaled via LOCA downscaling. This, together with the fact that daily vertically-resolved humidity and wind data needed to compute IVT were made available only for the first run of most GCMs for download from the World Climate Research Programme^73^, dictated our choice of GCM realizations. For two of the GCMs, however – CanESM2 and MIROC5, the requisite data are available for five realizations each. To partially validate our choice of the Real-5 GCMs, we applied our AR detection methodology and AR catalog validation algorithm to 4 additional realizations (r2i1p1, r3i1p1, r4i1p1, r5i1p1) available for CanESM2 (Real-5 category) and MIROC5 (Other-11 category), each. We assessed the realism of these two GCMs, in all five available realizations each, with respect to their ability to simulate the AR landfalling climatology along the West Coast. The results from these additional realizations (not shown) are much more similar to the first realization from the respective model than they are to results from other GCMs. We therefore conclude that our choice of Real-5 GCMs is unlikely to have been affected by the choice of realization from each GCM’s ensemble.

### Testing significance via bootstrap

Real-5 results showing reduction in uncertainty in projected AR event frequency compared to projected full ensemble of all 16 GCMs (Table S1) were tested for statistical significance via bootstrap resampling^74^. This involved generating a distribution of standard deviations in the projected changes among 2000 random combinations (out of *C(5*,*16)* = 4,368 possible combinations) of 5 randomly selected models out of the full ensemble of 16 GCMs and comparing the Real-5 result to that bootstrapped distribution. We found that the standard deviation associated with the Real-5 AR landfall frequency changes corresponded to the 2^nd^ percentile of the bootstrapped distribution, conservatively concluding that the reduction in uncertainty was statistically significant with 95% confidence.

We applied a similar approach to testing whether the ensemble average trend in total precipitation projected by the Real-5 GCMs (Fig. 4a and Table S5) were significant with respect to a distribution of such trends as projected by 2000 randomly selected 5-member ensembles, i.e. bootstrapped distribution. The Real-5 trend corresponded to the 96^th^ percentile of the bootstrapped distribution of trends, prompting us to conclude that it was not significant in a two-tailed test with 95% confidence. Specifically, although significant as trends go, we cannot confidently rule out the possibility that it could have resulted from random sampling of GCMs.

### Polynomial trend fitting and removal

In order to assess changes in natural variability in projected downscaled precipitation (Fig. 3), we removed a polynomial (2^nd^ order) trend^75^ fitted to the full 150-year record of LOCA-downscaled water-year precipitation in each of the Real-5 models and at each 6 × 6 km grid cell. For this purpose, the R function “poly” was used.

## Supplementary information

Supplementary Materials
