# Supplementary material for: Precipitation regime change in Western North America: The role of Atmospheric Rivers
Source: Sci Rep. 2019 Jul 9;9:9944. doi: 10.1038/s41598-019-46169-w (PMC6617450; doi:10.1038/s41598-019-46169-w)
Supplement: Supplementary file 1 — Supplementary Materials [file 41598_2019_46169_MOESM1_ESM.pdf]

SUPPLEMENTARY INFORMATION FOR

Precipitation regime change in Western North America: The role of Atmospheric Rivers

Alexander Gershunov*[1,2], Tamara Shulgina[1,2], Rachel E.S. Clemesha[2], Kristen Guirguis[1,2], David W. Pierce[2], Michael D. Dettinger[3], David A. Lavers[4], Daniel R. Cayan[2], Suraj D. Polade[5], Julie Kalansky[1,2] and F. Martin Ralph[1,2]

[1]Center for Western Weather and Water Extremes (CW3E),
[2]Climate, Atmospheric Science and Physical Oceanography (CASPO),
Scripps Institution of Oceanography, University of California San Diego, USA

[3]United States Geologic Survey, Carson City, Nevada, USA

[4]European Centre for Medium-Range Weather Forecasts (ECMWF), Reading, UK

[5]Finnish Meteorological Institute, Helsinki, Finland

Submitted to Nature Scientific Reports, March 6, 2019

*Correspondence to **sasha@ucsd.edu**

Table S1. CMIP5 GCMs used to detect ARs landfalling at the North American West Coast over historical and projected periods. Asterisk (*) marks GCMs where 10m data were substituted for 1000 mb data (see the text for details). GCMs selected for California Water resources planning[57] are indicated as "CalWat". The most realistic five models (Real-5) are highlighted in an italic font.

#	Model	Institution	Latitude resolution	Longitude resolution
1	***ACCESS1.0**** *(CalWat)*	*CSIRO (Commonwealth Scientific and Industrial Research Organization), and BOM (Bureau of Meteorology), Australia*	*1.25°*	*~1.88°*
2	***ACCESS1.3****	*CISRO and BOM, Australia*	*1.25°*	*~1.88°*
3	**BCC-CSM1.1**	Beijing Climate Center, China Meteorological Administration, China	~2.79°	~2.81°
4	***CanESM2*** *(CalWat)*	*Canadian Centre for Climate Modelling and Analysis, Canada*	*~2.79°*	*~2.81°*
5	***CNRM-CM5*** *(CalWat)*	*Centre National de Recherches Meteorologiques / Centre Europeen de Recherche et Formation Avancees en Calcul Scientifique, France*	*~1.4°*	*~1.41°*
6	***GFDL-CM3**** *(CalWat)*	*Geophysical Fluid Dynamics Laboratory, United States*	*2°*	*2.5°*
7	**GFDL-ESM2G***	Geophysical Fluid Dynamics Laboratory, United States	~2.02°	2.5°
8	**GFDL-ESM2M*** (missing 1995-2005)	Geophysical Fluid Dynamics Laboratory, United States	~2.02°	2.5°
9	**HadGEM2-CC** (CalWat)	Met Office Hadley Centre, United Kingdom	1.25°	~1.88°
10	**Inmcm4**	Institute for Numerical Mathematics, Russia	1.5°	2°
11	**IPSL-CM5A-LR***	Institut Pierre-Simon Laplace, France	~1.89°	3.75°
12	**IPSL-CM5A-MR***	Institut Pierre-Simon Laplace, France	~1.27°	2.5°
13	**MIROC5*** (CalWat)	Atmosphere and Ocean Research Institute (The University of Tokyo), National Institute for Environmental Studies, and Japan Agency for Marine-Earth Science and Technology, Japan	~1.4°	~1.41°
14	**MIROC-ESM***	Japan Agency for Marine-Earth Science and Technology, Atmosphere and Ocean Research Institute (The University of Tokyo), and National Institute for Environmental Studies, Japan	~2.79°	~2.81°

15	**MIROC-ESM-CHEM***	Japan Agency for Marine-Earth Science and Technology, Atmosphere and Ocean Research Institute (The University of Tokyo), and National Institute for Environmental Studies, Japan	~2.79°	~2.81°
16	**MRI-CGCM3***	Meteorological Research Institute, Japan	~1.12°	~1.13°

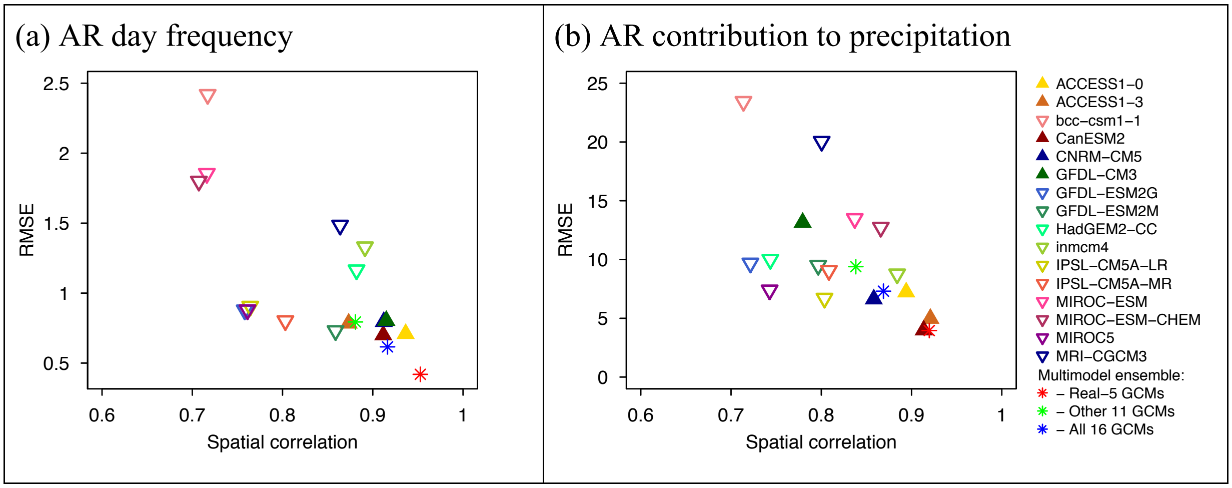

Figure S1. The agreement between each of the GCMs (triangles) and the SIO R1-based climatologies of (a) AR day frequency by month and landfalling latitude and (b) AR contribution to annual total precipitation estimated using LOCA-downscaled GCM precipitation[35] and gridded observed data[37]. Asterisks show the agreement between observed and multimodel ensemble average climatologies of all 16 GCMs (blue), Real-5 (red), and the Other 11 GCMs (green).

AR land-falling activity at West Coast

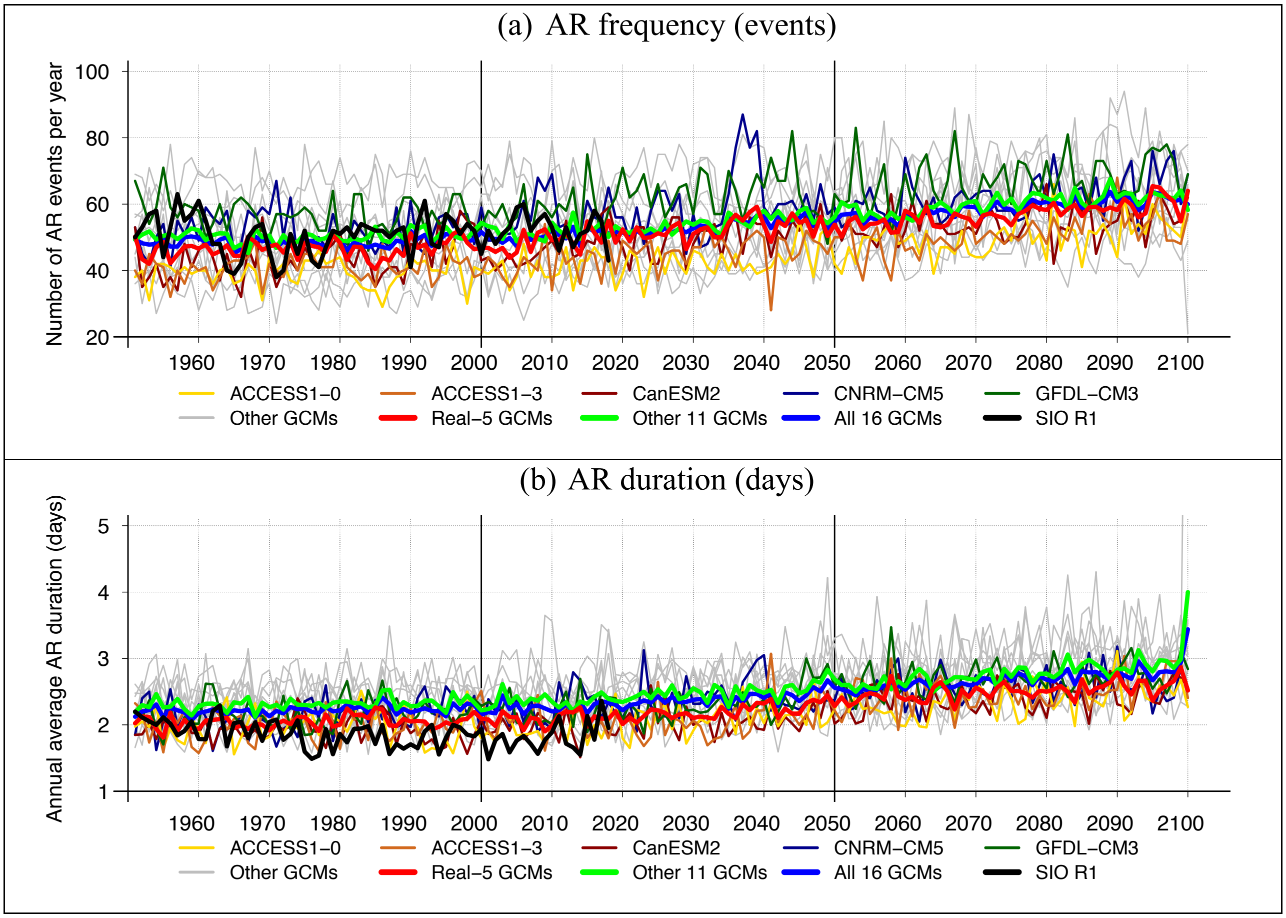

Figure S2. The annual number of AR events (a) hit the West Coast [20-60°N] and average duration of those events (b) in historical (1951-2005, left panel) and projected (2007-2100, right panel) epochs. Real-5 GCMs (CNRM-CM5, CanESM2, ACCESS1-0, ACCESS1-3 and GFDL-CM3) are plotted in thin colored lines, while other GCMs are outlined in gray. Thick curves represent the ensemble averages of Real-5 GCMs (red), the other 11 GCMs (green) and the full ensemble of 16 GCMs (blue). Thick black curve delineates the average annual behavior of AR frequency (a) and duration (b), which is based on NCEP/NCAR Reanalysis data.

AR statistics at West Coast

Table S2. Comparative statistics for each of the GCM AR records over the historical (1951 – 2000) and RCP8.5 (2051 – 2100) projected periods. "*" indicates GCMs that did not report data at 1000 Mb (see Method section for handling details). GCMs selected for California Water resources planning are marked as "CalWat". The table rows with an italic font highlight the statistics of Real-5 GCMs, the average statistics of which is summarized toward the end of the table. The average statistics for the other 11 GCMs and the full ensemble of 16 GCMs is shown as well. Change in AR characteristics for the future according to RCP 8.5 scenario is estimated in percentage (%) relative to the correspond AR characteristics estimated for the historical (1951-2000) period.

**Model**	**Average maximum IVT per AR event (kg/m/s)**		**Change in average maximum IVT per AR event (%)**	**Frequency of AR events per year**		**Change (%) in AR event frequency**	**Duration of AR events (days)**		**Change (%) in AR event duration**
	**1951-2000**	**2051-2100**		**1951-2000**	**2051-2100**		**1951-2000**	**2051-2100**	
ACCESS1.0* *(CalWat)*	*365*	*400*	*10 %*	*39*	*49*	*26 %*	*2*	*2.4*	*19 %*
ACCESS1.3*	*370*	*411*	*11 %*	*41*	*51*	*25 %*	*2*	*2.5*	*23 %*
**BCC-CSM1.1**	444	485	9 %	63	68	8 %	2.6	3	17 %
CanESM2 *(CalWat)*	*402*	*450*	*12 %*	*44*	*54*	*23 %*	*1.9*	*2.4*	*25 %*
CNRM-CM5 *(CalWat)*	*418*	*461*	*10 %*	*52*	*62*	*20 %*	*2.1*	*2.6*	*20 %*
GFDL-CM3* *(CalWat)*	*404*	*459*	*14 %*	*57*	*67*	*19 %*	*2.2*	*2.7*	*24 %*
**GFDL-ESM2G***	394	425	8 %	47	62	31 %	2.1	2.4	13 %
**GFDL-ESM2M*** (missing 1995-2005)	395	428	8 %	50	63	26 %	2	2.3	14 %
**HadGEM2-CC** (CalWat)	383	430	12 %	35	50	43 %	2	2.6	33 %
**Inmcm4**	424	445	5 %	54	57	5 %	2.6	3	14 %
**IPSL-CM5A-LR***	408	464	14 %	37	45	22 %	2.3	3	29 %
**IPSL-CM5A-MR***	415	457	10 %	43	56	31 %	2.3	2.7	22 %
**MIROC5*** (CalWat)	398	419	5 %	44	58	32 %	2.1	2.4	12 %
**MIROC-ESM***	395	431	9 %	53	65	22 %	2.3	2.8	21 %
**MIROC-ESM-CHEM***	394	429	9 %	52	62	19 %	2.3	3	30 %
**MRI-CGCM3***	435	463	6 %	67	74	10 %	2.5	2.8	15 %
**Real-5 GCMs average**	**392**	**436**	**11 %**	**47**	**57**	**21 %**	**2**	**2.5**	**22 %**
**Other 11 GCMs average**	**408**	**443**	**9 %**	**49**	**59**	**20 %**	**2.3**	**2.8**	**19 %**
**All 16 GCM average**	**403**	**441**	**10 %**	**48**	**58**	**21 %**	**2.2**	**2.7**	**21 %**
**SIO-R1**	372	-	-	50	-	-	1.9	-	-

Trends in AR land-falling activity at West Coast

Table S3. Linear trends in average annual AR event frequency (middle panel), AR event duration (right panel) and maximum IVT per AR event (left panel) at the West Coast [20-60°N] during the historical (1951-2000), current (2001-2050) and projected (2051-2100) periods as well as entire 21st century (2001-2100). Values in brackets show these trends relative to the historical average. Statistically significant changes are in red if positive, green – if negative. The table rows with an italic font denoted Real-5, the rows with a bold font – GCM ensembles.

Global Climate Model	Trends in the maximum AR IVT (kg/m/s, percent) fitted over the study period				Trends in number of AR events (event, percent) fitted over the study periods				Trends in average AR duration (days, percent) fitted over the study period			
	1951-2000	2001-2050	2051-2100	2001-2100	1951-2000	2001-2050	2051-2100	2001-2100	1951-2000	2001-2050	2051-2100	2001-2100
ACCESS1-0	*-2(0%)*	*+15(4%)*	*+26(7%)*	*+47(12%)*	*<+1(1%)*	*+6(14%)*	*+9(18%)*	*+16(34%)*	*-0.04(2%)*	*+0.33(16%)*	*+0.34(14%)*	*+0.63(29%)*
ACCESS1-3	*+9(2%)*	*+23(6%)*	*+6(2%)*	*+59(15%)*	*+2(4%)*	*+7(17%)*	*+11(21%)*	*+14(29%)*	*-0.11(5%)*	*+0.22(10%)*	*+0.25(10%)*	*+0.73(32%)*
**bcc-csm1-1**	-16(4%)	+34(7%)	+18(4%)	+51(11%)	+2(3%)	-2(3%)	+5(7%)	+7(11%)	+0.13(5%)	+0.54(20%)	+0.24(8%)	+0.63(22%)
CNRM-CM5	*+7(2%)*	*+34(8%)*	*+25(5%)*	*+46(10%)*	*+2(3%)*	*+9(16%)*	*+10(16%)*	*+10(17%)*	*+0.23(11%)*	*+0.23(10%)*	*+0.11(4%)*	*+0.41(17%)*
CanESM2	*-4(1%)*	*+26(6%)*	*+34(7%)*	*+68(16%)*	*+8(18%)*	*+10(21%)*	*+6(10%)*	*+12(23%)*	*-0.05(3%)*	*+0.19(9%)*	*+0.26(11%)*	*+0.68(31%)*
GFDL-CM3	*+8(2%)*	*+32(8%)*	*+43(10%)*	*+79(18%)*	*-4(7%)*	*+6(10%)*	*+7(10%)*	*+12(18%)*	*+0.15(7%)*	*+0.37(16%)*	*+0.17(6%)*	*+0.75(30%)*
**GFDL-ESM2G**	+19(5%)	+9(2%)	+48(11%)	+39(9%)	-1(2%)	+8(15%)	+14(23%)	+21(36%)	+0.16(8%)	+0.09(4%)	+0.33(14%)	+0.22(10%)
**GFDL-ESM2M**	+8(2%)	+23(6%)	+15(3%)	+35(8%)	-6(12%)	+9(16%)	+8(13%)	+19(32%)	-0.74(39%)	+1.47(74%)	+0.19(8%)	+0.9(42%)
**HadGEM2-CC**	+3(1%)	+22(6%)	+37(9%)	+68(16%)	+1(3%)	+12(29%)	+3(5%)	+18(39%)	+0.08(4%)	+0.22(11%)	+0.57(23%)	+0.79(35%)
**inmcm4**	-7(2%)	-2(1%)	+25(6%)	+28(6%)	+4(8%)	+6(12%)	0(0%)	+4(7%)	-0.07(3%)	-0.1(4%)	+0.32(11%)	+0.48(17%)
**IPSL-CM5A-LR**	-20(5%)	+18(4%)	+81(17%)	+84(19%)	+2(7%)	+2(5%)	+1(3%)	+8(19%)	+0.02(1%)	+0.37(15%)	+0.81(28%)	+1.03(38%)
**IPSL-CM5A-MR**	-7(2%)	+32(7%)	+31(7%)	+52(12%)	<+1(1%)	+7(16%)	+7(13%)	+18(34%)	+0.03(1%)	+0.35(15%)	+0.25(9%)	+0.62(24%)
**MIROC5**	+5(1%)	+17(4%)	+13(3%)	+36(9%)	+1(2%)	+11(22%)	+8(14%)	+20(38%)	-0.02(1%)	+0.2(10%)	+0.28(12%)	+0.46(20%)
**MIROC-ESM**	+10(3%)	+25(6%)	+31(7%)	+51(12%)	<+1(1%)	+2(4%)	+8(12%)	+17(28%)	+0.15(6%)	+0.63(25%)	+0.45(16%)	+0.69(25%)
**MIROC-ESM-CHEM**	+8(2%)	+36(9%)	+37(9%)	+60(15%)	+1(1%)	+8(15%)	+13(20%)	+13(22%)	+0.13(5%)	+0.45(18%)	+0.29(10%)	+0.97(35%)
**MRI-CGCM3**	0(0%)	+21(5%)	+29(6%)	+46(10%)	+1(1%)	+5(8%)	+5(6%)	+10(13%)	+0.05(2%)	+0.2(8%)	+0.37(13%)	+0.56(21%)
**Real-5**	**+4(1%)**	**+26(6%)**	**+27(6%)**	**+60(14%)**	**+2(3%)**	**+8(15%)**	**+8(15%)**	**+13(23%)**	**+0.08(4%)**	**+0.27(12%)**	**+0.23(9%)**	**+0.64(28%)**
**11GCMs**	**0(0%)**	**+20(5%)**	**+35(8%)**	**+51(12%)**	**+1(3%)**	**+6(11%)**	**+6(10%)**	**+13(23%)**	**+0.07(3%)**	**+0.29(12%)**	**+0.41(15%)**	**+0.65(25%)**
**16GCMs**	**+1(0%)**	**+26(6%)**	**+32(7%)**	**+56(13%)**	**0(0%)**	**+7(13%)**	**+6(11%)**	**+13(23%)**	**+0.02(1%)**	**+0.34(15%)**	**+0.34(13%)**	**+0.66(26%)**
**R1**	**-7(2%)**	###	###	###	**<-1(1%)**	###	###	###	-0.27(14%)	###	###	###

AR land-falling activity in California

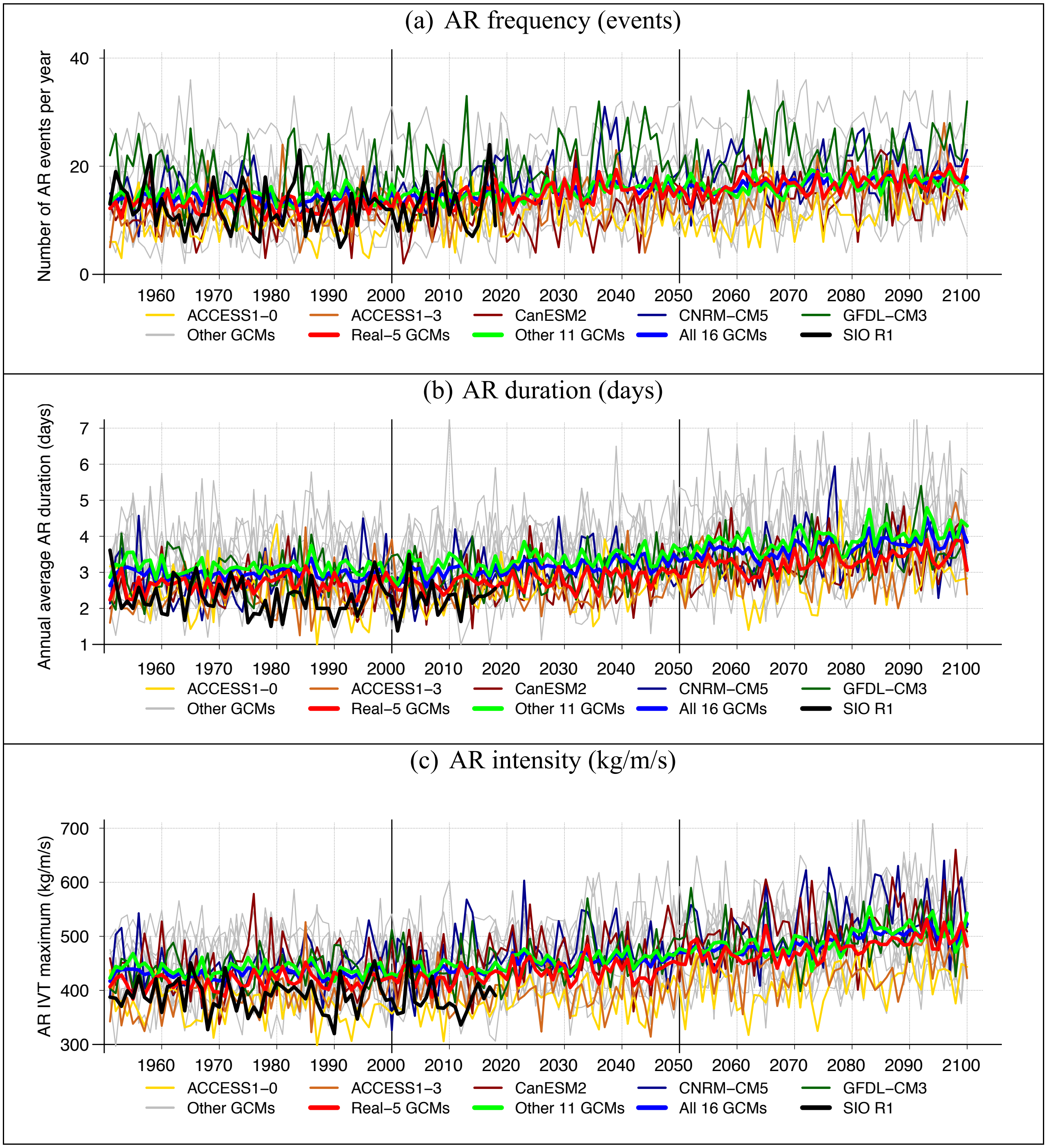

Figure S3. The annual number AR events (a) in **California**, average duration (b) and maximum IVT magnitude (c) of those events in historical (1951-2005, left) and projected (2007-2100, right) epochs. Real-5 GCMs (CNRM-CM5, CanESM2, ACCESS1-0, ACCESS1-3 and GFDL-CM3) are plotted in thin colored lines, while other GCMs are outlined in gray. Thick curves represent the ensemble averages of Real-5 GCMs (red), the other 11 GCMs (green) and the full ensemble of 16 GCMs (blue). Thick black curve delineates the average annual behavior of AR

day frequency (a), AR event duration (b) and intensity (c), which is based on NCEP/NCAR Reanalysis data.

Trends in AR land-falling activity in California

Table S4. Linear trends in average annual AR event frequency (middle panel), AR event duration (right panel) and maximum IVT per AR event (left panel) in California during the historical (1951-2000), current (2001-2050) and projected (2051-2100) periods as well as entire 21st century (2001-2100). Values in brackets show these trends relative to the historical average. Statistically significant changes are in red if positive, green – if negative. The table rows with an italic font denoted Real-5, the rows with a bold font – GCM ensembles.

Global Climate Model	Trends in the maximum AR IVT (kg/m/s, percent) fitted over the study period				Trend in number of AR events (event, percent) fitted over the study period				Trends in average AR duration (days, percent) fitted over the study period			
	1951-2000	2001-2050	2051-2100	2001-2100	1951-2000	2001-2050	2051-2100	2001-2100	1951-2000	2001-2050	2051-2100	2001-2100
ACCESS1-0	*-26 (6 %)*	*+16(4%)*	*+45 (11 %)*	*+59(15%)*	*-2(20%)*	*<+1(2%)*	*+5(47%)*	*+2(22%)*	*-0.53(22%)*	*+0.58(23%)*	*+0.61(21%)*	*+0.79(29%)*
ACCESS1-3	*+42 (1 %)*	*-4(1%)*	*+29 (7 %)*	*+69(16%)*	*+3(26%)*	*+3(22%)*	*+5(33%)*	*+4(43%)*	*+0.62(26%)*	*+0.11(4%)*	*+0.38(12%)*	*+1.08(38%)*
bcc-csm1-1	-19 (4 %)	+80(16%)	+54 (10 %)	+95(18%)	+1(5%)	+2(27%)	>-1(2%)	+2(8%)	+0.1(3%)	+0.64(16%)	+0.5(11%)	+1.37(32%)
CNRM-CM5	*+16 (4 %)*	*+25(5%)*	*+65 (13%)*	*+98(20%)*	*-0(3%)*	*+3(17%)*	*+3(17%)*	*+4(21%)*	*+0.35(13%)*	*+0.52(17%)*	*+0.26(7%)*	*+0.86(26%)*
CanESM2	*+7 (2 %)*	*+28(6%)*	*+50 (10%)*	*+105(22%)*	*+0(4%)*	*+3(27%)*	*+1(4%)*	*+6(46%)*	*-0.23(9%)*	*+0.17(6%)*	*+0.55(16%)*	*+1.16(38%)*
GFDL-CM3	*+17 (4 %)*	*+36(8%)*	*+22 (5 %)*	*+82(17%)*	*-4(21%)*	*+5(23%)*	*+2(10%)*	*+4(21%)*	*+0.3(10%)*	*+0.23(8%)*	*+0.18(5%)*	*+0.91(28%)*
GFDL-ESM2G	-4 (1 %)	+10(2%)	+84 (18 %)	+63(14%)	-1(5%)	+4(22%)	+2(28%)	+3(15%)	+0.12(5%)	-0.01(0%)	+0.7(22%)	+0.42(14%)
GFDL-ESM2M	+6 (2 %)	+57(13%)	+27 (6 %)	+59(13%)	-2(9%)	<+1(1%)	+2(12%)	+3(16%)	0(0%)	+0.87(30%)	+0.45(15%)	+0.5(17%)
HadGEM2-CC	+11 (3 %)	+56(14%)	+72 (16 %)	+117(28%)	+1(17%)	+5(60%)	+0(1%)	+5(52%)	+0.19(9%)	+0.49(21%)	+1.03(36%)	+1.28(49%)
inmcm4	+7 (2 %)	-10(2%)	+43 (8 %)	+64(13%)	+1(5%)	<+1(2%)	-2(10%)	+2(16%)	+0.03(1%)	-0.22(6%)	+0.44(10%)	+1.16(28%)
IPSL-CM5A-LR	-6 (1 %)	+47(10%)	+137(26%)	+141(28%)	0(0%)	<+1(1%)	+3(19%)	+6(43%)	-0.07(2%)	+1.03(31%)	+1.21(31%)	+1.59(44%)
IPSL-CM5A-MR	-16 (4 %)	+65(13%)	+43 (8 %)	+63(13%)	+1(4%)	+5(23%)	+1(5%)	+8(46%)	-0.37(13%)	+0.75(24%)	+0.65(18%)	+0.89(27%)
MIROC5	-13 (3 %)	-2(0%)	- 29 (6 %)	+43(10%)	-0(1%)	+2(15%)	+5(30%)	+5(34%)	-0.26(10%)	+0.46(17%)	+0.06(2%)	+1.13(37%)
MIROC-ESM	+6 (2 %)	+20(5%)	+30 (8 %)	+41(9%)	+1(12%)	-1(6%)	+3(25%)	+3(24%)	-0.07(2%)	+0.94(27%)	+0.52(13%)	+0.96(26%)
MIROC-ESM-CHEM	-14 (3 %)	-1(0%)	+38 (8 %)	+69(15%)	-1(5%)	-1(5%)	+4(33%)	<+1(3%)	-0.71(22%)	+0.05(1%)	+0.21(5%)	+1.41(36%)
MRI-CGCM3	-9 (2 %)	+54(11%)	+43 (8 %)	+95(19%)	-1(4%)	+6(24%)	>-1(1%)	+5(18%)	+0.02(1%)	+0.29(8%)	+0.5(12%)	+1.03(27%)
**Real-5**	**+11 (3 %)**	**+20(5%)**	**+42 (9 %)**	**+83(18%)**	**>-1(4%)**	**+3(19%)**	**+3(20%)**	**+5(30%)**	**+0.1(4%)**	**+0.32(12%)**	**+0.4(12%)**	**+0.96(32%)**
**11GCM-NSBL**	**-4 (-1%)**	**+28(6%)**	**+53 (11%)**	**+81(17%)**	**<+1(1%)**	**+2(16%)**	**+2(12%)**	**+3(22%)**	**-0.07(2%)**	**+0.41(12%)**	**+0.57(17%)**	**+1.15(32%)**
**16GCM-NSBL**	**+1 (0 %)**	**+32(7%)**	**+48(10 %)**	**+83(18%)**	**>-1(2%)**	**+3(17%)**	**+2(12%)**	**+4(23%)**	**0(0%)**	**+0.42(13%)**	**+0.54(15%)**	**+1.06(31%)**
SIO-R1	-6 (2 %)	###	###	###	**-2(15%)**	**###**	**###**	**###**	-0.21(9%)	###	###	###

Average climatology of Real-5 AR activity by month and land-falling latitude

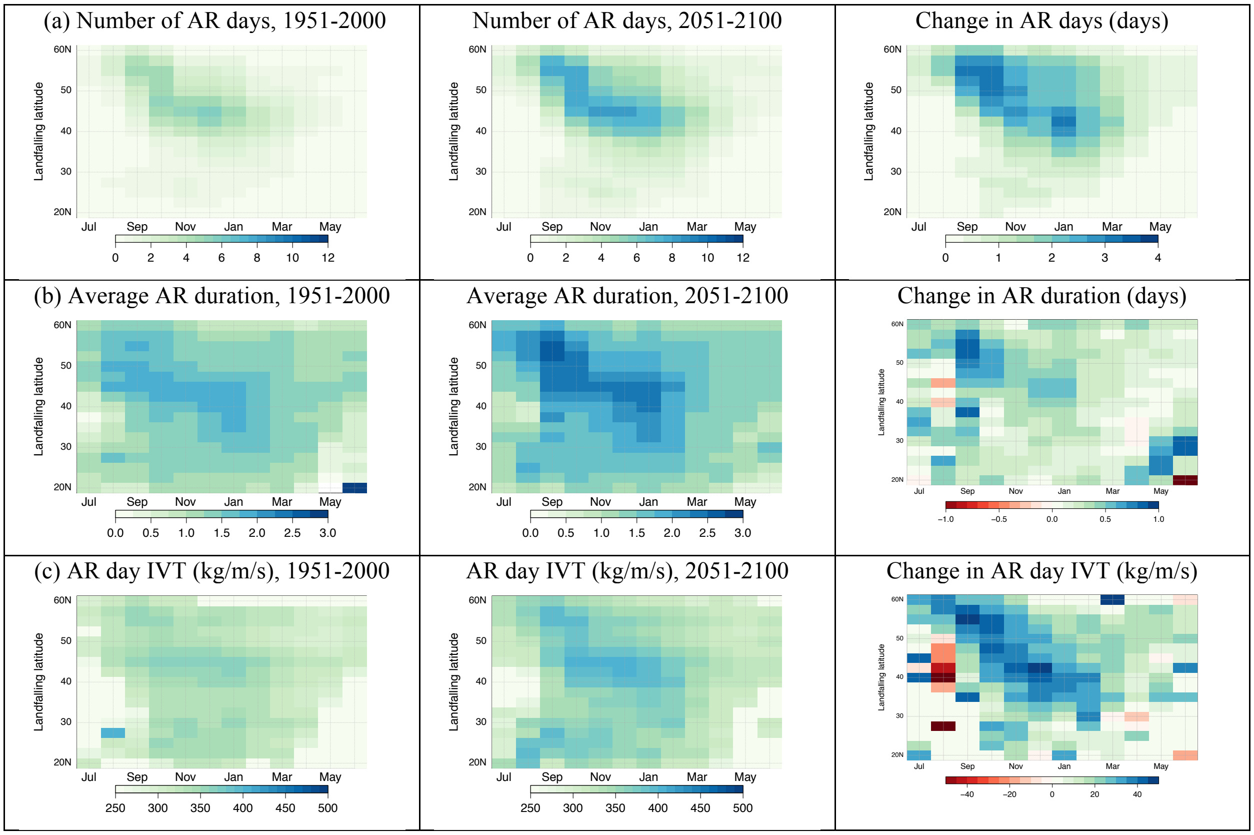

Figure S4. Real-5 average monthly climatology of (a-panel, horizontally) land-falling AR day counts, (b-panel, horizontally) average local land-falling duration and (c-panel, horizontally) daily average IVT (kg/m/s) associated with AR conditions at the North America west coast during 1951-2000 (left panel, vertically) and 2051-2100 (middle panel, vertically) time periods. Right panel shows the change (days) in the number of AR days (days), AR duration (days) and IVT (kg/m/s) projected for the future relative to the correspond AR characteristics estimated for the past (1951-2000) by month and latitude.

Daily average precipitation intensity

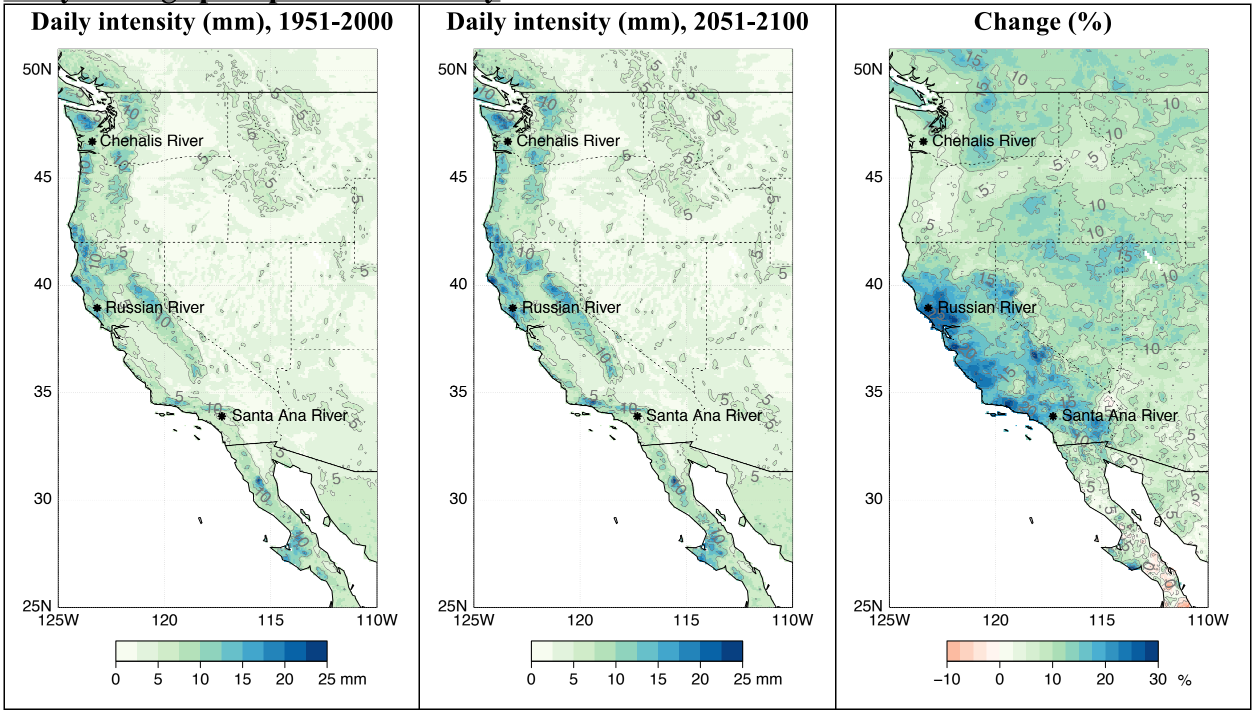

Figure S5. Real-5 LOCA GCM ensemble average of daily precipitation intensity during historical (1951-2000, left) and projected (2051-2100, middle) water years (July-June) and (right) the change (%) of daily precipitation intensity over the projected period, relative to the historical period, using RCP8.5 forcing scenarios.

Annual precipitation frequency and AR contribution

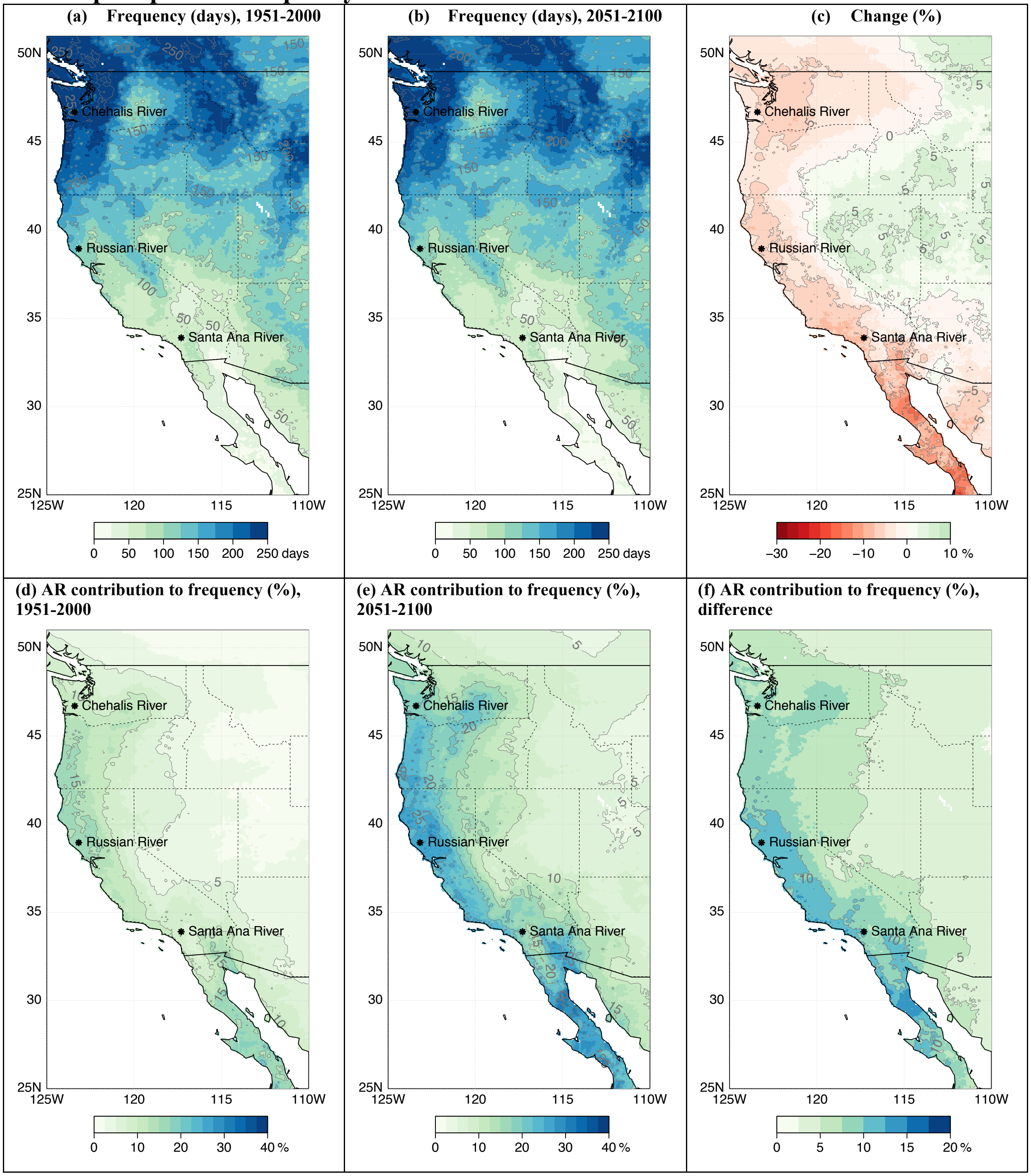

Figure S6. Real-5 LOCA GCM ensemble average of (top row, a-b) annual average precipitation frequency and (bottom row, d-e) contribution of AR-related precipitation to total precipitation frequency during the historical (1951-2000, left column) and projected (2051-2100, middle column) water years (July-June). The change (c) of total precipitation frequency during the second half of 21st century is expressed in percent (%) relative to the second half of 20th century, using RCP8.5 forcing scenarios. The change (f) in AR contribution to total precipitation

frequency is computed as difference between projected and historical AR contribution climatologies.

Annual precipitation sum and AR contribution

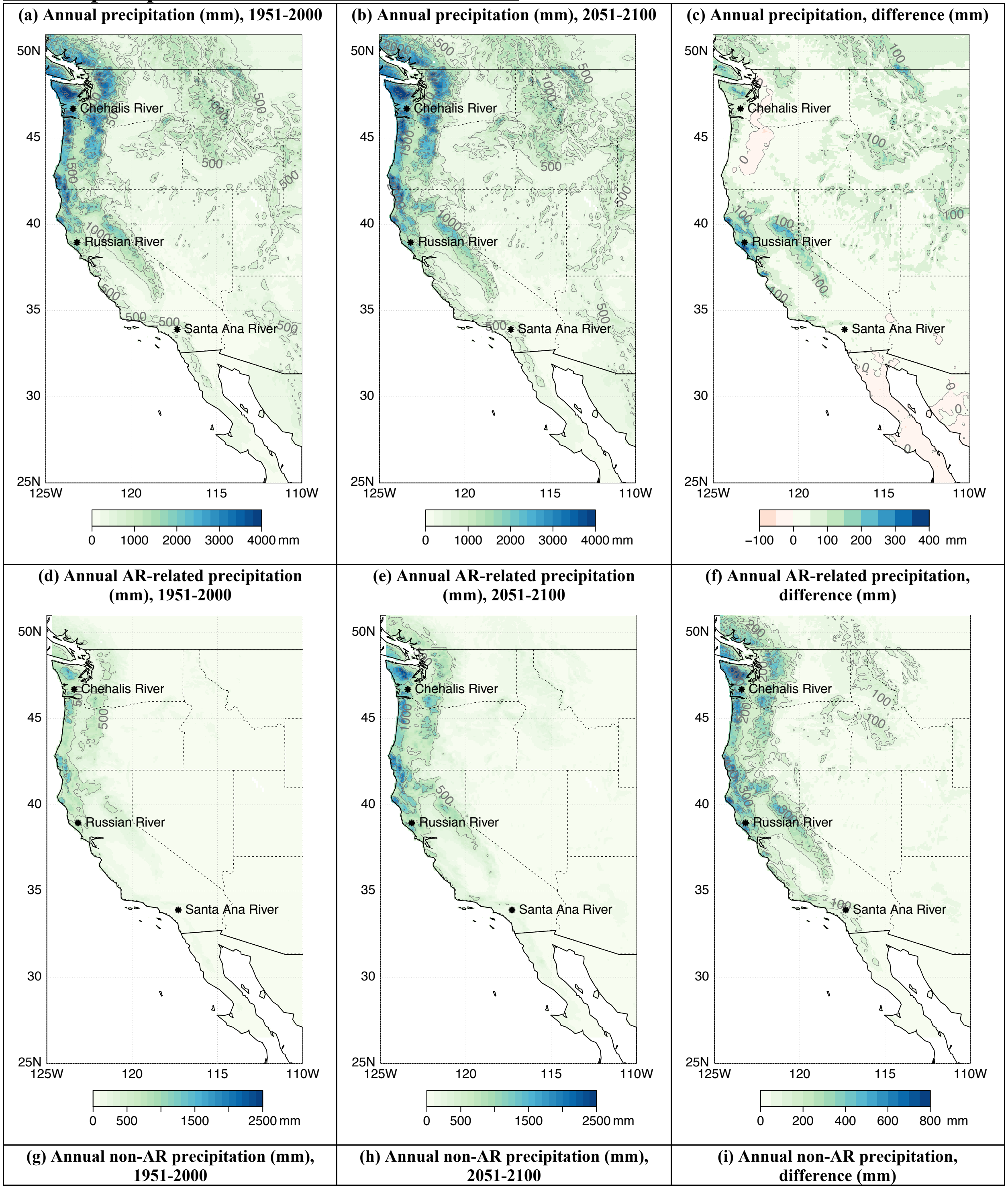

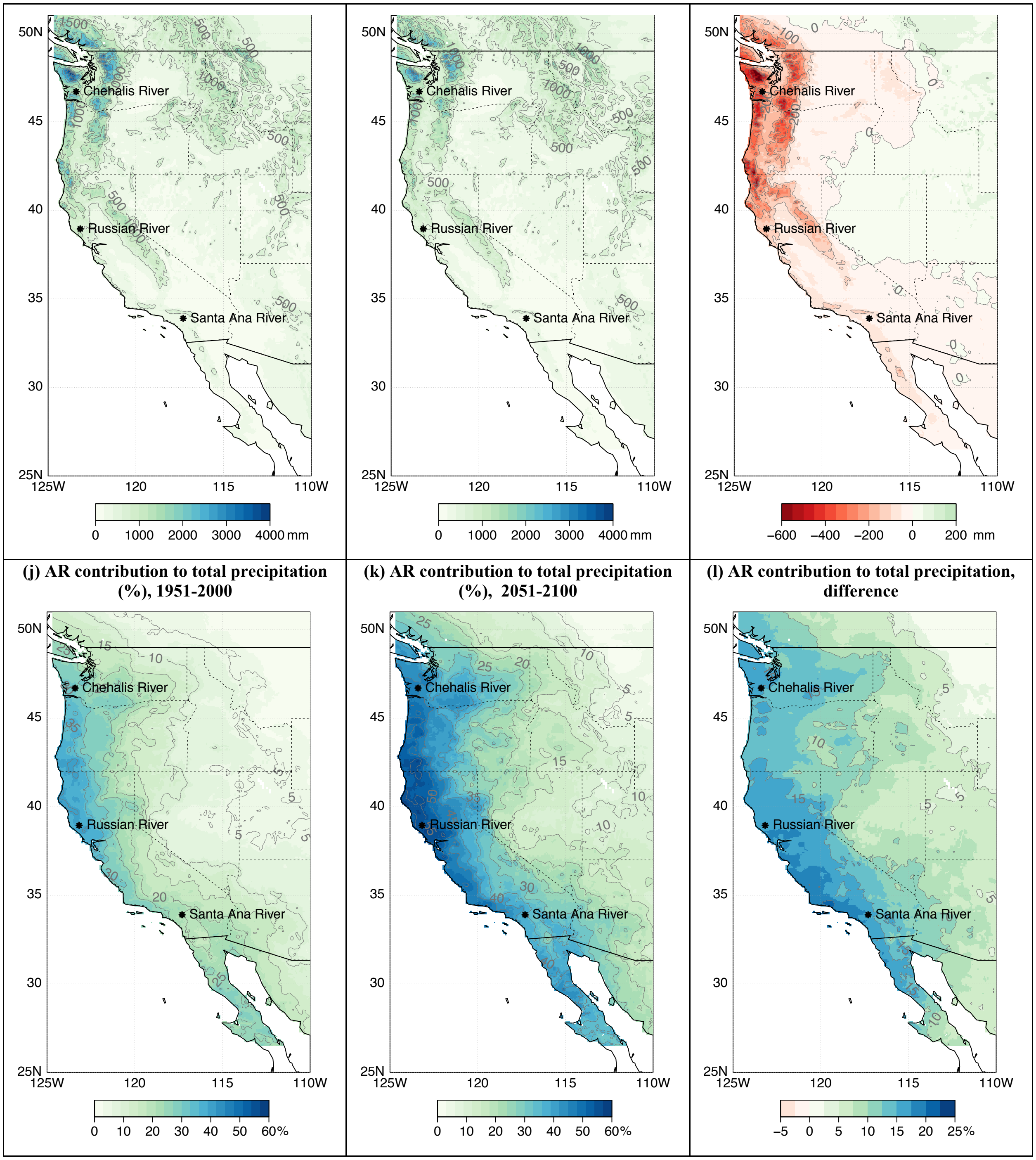

Figure S7. Real-5 LOCA GCM ensemble average of (top row, a-b) annual precipitation, (second row, d-e) AR precipitation, (third row, g-h) non-AR precipitation and the contribution (bottom row, j-k) of AR precipitation to total precipitation sum during historical (1951-2000, left column) and projected (2051-2100, middle column) water years (July-June). The change (c) in annual precipitation, (f) AR precipitation, (i) non-AR precipitation and (l) AR precipitation contribution during the second half of 21st century is computed as difference between projected climatologies using RCP8.5 forcing scenarios and historical climatologies over the second half of 20th century, respectively.

Table S5. Linear change in annual precipitation in California during the 50-year period of historical (1951-2000) and future projected (2007-2100) time periods. Values in brackets show these changes relative to historical and future 50-year average, respectively. Statistically significant changes are in red if they are positive, in green – if negative. The table rows with an italic font denoted Real-5, the rows with a bold font – GCM ensembles.

Global Climate Model	Trends in annual all precipitation (mm, percent) over the study period				Trends in annual AR precipitation (mm, percent) over the study period				Trends in annual non-AR precipitation (mm, percent) over the study period			
	1951-2000	2001-2050	2051-2100	2001-2100	1951-2000	2001-2050	2051-2100	2001-2100	1951-2000	2001-2050	2051-2100	2001-2100
ACCESS1-0	+13 (2 %)	-49(8%)	+59 (11 %)	-117(20%)	-22 (18 %)	+41(27%)	+113(62%)	+78(46%)	+35 (7 %)	-90(19%)	-54 (15 %)	-195(47%)
ACCESS1-3	+102(17%)	+81(13%)	+212(31%)	+145(22%)	+63 (37 %)	+57(28%)	+214(74%)	+198(80%)	+40 (9 %)	23(6%)	-2 (1 %)	-53(13%)
Bcc-csm1-1	+45 (7 %)	+92(16%)	-210 (36%)	-25(4%)	+37 (11 %)	+124(34%)	-127 (30%)	+89(23%)	+7 (3 %)	-32(14%)	-84 (50 %)	-114(59%)
CNRM-CM5	-29 (5 %)	+108(16%)	+48 (6 %)	+167(23%)	+11 (5 %)	+119(41%)	+120(28%)	+226(74%)	-40 (10 %)	-11(3%)	-72 (21 %)	-99(27%)
CanESM2	-48 (8 %)	+136(21%)	+99 (12 %)	+334(46%)	+9 (5 %)	+125(53%)	+100(24%)	+335(102%)	-57 (13 %)	11(3%)	0 (0 %)	-1(0%)
GFDL-CM3	-20 (3 %)	-41(7%)	-153(25 %)	-57(9%)	-14 (5 %)	+32(10%)	-30 (7 %)	+131(36%)	-6 (2 %)	-73(25%)	-123(63 %)	-188(77%)
GFDL-ESM2G	-57 (9 %)	+135(22%)	+23 (4 %)	-24(4%)	0 (0 %)	+104(38%)	+138(47%)	+91(32%)	-57 (14 %)	+31(9%)	-115(40 %)	-115(36%)
GFDL-ESM2M	-109(18%)	+4(1%)	-36(7%)	-99(18%)	-57(24%)	53(19%)	+51(18%)	+38(14%)	-35(9%)	-60(19%)	-87(35%)	-151(53%)
HadGEM2-CC	-1 (0 %)	+73(12%)	-63 (11 %)	-15(3%)	+13 (20 %)	+91(78%)	+31 (16 %)	+141(91%)	-14 (3 %)	-19(4%)	-94 (23 %)	-156(35%)
inmcm4	-53 (12 %)	-201(33%)	-93 (14 %)	31(5%)	13 (5 %)	-125(46%)	-5 (1 %)	+129(40%)	-66 (19 %)	-75(23%)	-88 (30 %)	-98(31%)
IPSL-CM5A-LR	-5(1%)	-78(13%)	+208(32%)	+101(16%)	-68(32%)	+21(8%)	+275(69%)	+291(89%)	+63(16%)	-99(29%)	-67(27%)	-190(64%)
IPSL-CM5A-MR	+2 (0 %)	+74(12%)	-21 (3 %)	43(7%)	-39 (15 %)	+189(60%)	+85 (20 %)	+244(65%)	+42 (11 %)	-115(38%)	-106(51 %)	-201(79%)
MIROC5	+22 (4 %)	-34(6%)	-31 (6 %)	-53(9%)	1 (0 %)	+24(12%)	+41 (16 %)	+85(37%)	+21 (5 %)	-58(15%)	-72 (23 %)	-138(40%)
MIROC-ESM	+58 (9 %)	-93(16%)	-9 (2 %)	-138(26%)	+60 (22 %)	-42(15%)	+88 (30 %)	+44(16%)	-2 (1 %)	-52(18%)	-97 (49 %)	-182(74%)
MIROC-ESM-CHEM	-64 (10 %)	-127(22%)	-55 (11 %)	-206(38%)	-4 (2 %)	-34(11%)	+36 (12 %)	-13(4%)	-60 (17 %)	-93(33%)	-91 (50 %)	-193(83%)
MRI-CGCM3	-35 (6 %)	+156(25%)	-63 (9 %)	+94(14%)	-29 (8 %)	+143(35%)	-57 (11 %)	+172(37%)	-6 (3 %)	13(6%)	-6 (4 %)	-78(39%)
**5GCM-NSBL**	***+4 (1 %)***	***+47(7%)***	***+53 (8 %)***	***+94(14%)***	***9 (5 %)***	***+75(31%)***	***+103(30%)***	***+202(69%)***	***-6 (1 %)***	***-28(7%)***	***-50 (15 %)***	***-107(29%)***
**11GCM-NSBL**	***-18 (3 %)***	***+0(0%)***	***-32 (5 %)***	***-27(4%)***	***12 (5 %)***	***+43(15%)***	***+51 (15 %)***	***+118(38%)***	***-20 (5 %)***	***-62(19%)***	***-82 (33 %)***	***-155(55%)***
**16GCM-NSBL**	***-11 (2 %)***	***+15(2%)***	***-5 (1 %)***	***11(2%)***	***10 (4 %)***	***+51(19%)***	***+67 (20 %)***	***+144(47%)***	***-15 (4 %)***	***-56(16%)***	***-72 (26 %)***	***-143(47%)***
Livneh-2015	+ 42 (7 %)	###	###	###	-70 (32 %)	###	###	###	+112(28%)	###	###	###

Table S6. Linear change in annual precipitation at US West coast during the 50-year period of historical (1951-2000) and future projected (2007-2100) time periods. Values in brackets show these changes relative to historical and future 50-year average, respectively. Statistically significant changes are in red if they are positive, in green – if negative. The table rows with an italic font denoted Real-5, the rows with a bold font – GCM ensembles.

Global Climate Model	Trends in annual all precipitation (mm, percent) over the study period				Trend in annual AR precipitation (mm, percent) over the study period				Trends in annual non-AR precipitation (mm, percent) over the study period			
	1951-2000	2001-2050	2051-2100	2001-2100	1951-2000	2001-2050	2051-2100	2001-2100	1951-2000	2001-2050	2051-2100	2001-2100
ACCESS1-0	-33 (5 %)	0(0%)	+18 (3 %)	-20(3%)	-19 (28 %)	+30(37%)	+68 (58 %)	+78(78%)	-14 (3 %)	-31(6%)	-51 (11 %)	-97(20%)
ACCESS1-3	+34 (6 %)	+49(8%)	+43 (6 %)	+85(13%)	+12 (15%)	+41(42%)	+51 (33 %)	+109(86%)	22 (4 %)	+8(2%)	-9 (2 %)	-24(5%)
Bcc-csm1-1	-17 (3 %)	+31(5%)	-102 (17%)	-17(3%)	+1 (0 %)	+77(31%)	-5 (2 %)	+105(37%)	-19 (5 %)	-47(13%)	-97 (32 %)	-122(37%)
CNRM-CM5	-10 (2 %)	+22(3%)	+44 (6 %)	+89 (13%)	+13 (11%)	+59(37%)	+72 (32 %)	+130(67%)	-23 (5 %)	-37(8%)	-27 (6 %)	-41(9%)
CanESM2	-11 (2 %)	+102(16%)	+82 (11%)	+199(29%)	+13 (10%)	+71(47%)	+65 (27 %)	+166(85%)	-24 (5 %)	+31(6%)	+17 (3 %)	+33(7%)
GFDL-CM3	-32 (5 %)	+35(6%)	-38 (6 %)	+23(4%)	-2 (2 %)	+50(26%)	+42 (16 %)	+129(57%)	-30 (7 %)	-15(4%)	-80 (21 %)	-106(26%)
GFDL-ESM2G	-24 (4 %)	+89(14%)	+55 (9 %)	+25(4%)	+11 (9 %)	+58(35%)	+110(54%)	+99(54%)	-35 (7 %)	31(7%)	-56 (13 %)	-74(17%)
GFDL-ESM2M	-10(2%)	+11(2%)	-14(2%)	+25(4%)	+13(10%)	+54(34%)	+36(18%)	+93(51%)	-17(3%)	-42(10%)	-51(13%)	-68(16%)
HadGEM2-CC	-7 (1 %)	+25(4%)	+38 (6 %)	+20(3%)	+8 (19 %)	+58(79%)	+66 (53 %)	+106(108%)	-15 (3 %)	-32(6%)	-27 (5 %)	-87(17%)
inmcm4	-26 (4 %)	-57(9%)	0 (0 %)	+22(4%)	0 (0 %)	-20(12%)	+37 (16 %)	+85(43%)	-26 (6 %)	-37(9%)	-37 (9 %)	-63(15%)
IPSL-CM5A-LR	+44(7%)	-12(2%)	+85(14%)	+69(11%)	-23(22%)	+28(21%)	+143(65%)	+179(102%)	+67(13%)	-40(9%)	-58(14%)	-110(25%)
IPSL-CM5A-MR	-13 (2 %)	+16(3%)	-15 (2 %)	0(0%)	-15 (12 %)	+91(56%)	+67 (28 %)	+147(74%)	+1 (0 %)	-75(16%)	-81 (21 %)	-147(35%)
MIROC5	+30 (5 %)	+29(5%)	-96 (15 %)	+8(1%)	+6 (6 %)	+34(31%)	+2 (1 %)	+75(57%)	+24 (5 %)	-5(1%)	-98 (21 %)	-67(14%)
MIROC-ESM	+19 (3 %)	-4(1%)	+23 (4 %)	+13(2%)	+21 (11 %)	+21(10%)	+83 (31 %)	+118(50%)	-2 (0 %)	-24(6%)	-59 (17 %)	-105(27%)
MIROC-ESM-CHEM	-19 (3 %)	+26(4%)	-14 (2 %)	-39(6%)	+8 (4 %)	+66(30%)	+80 (31 %)	+95(40%)	-27 (6 %)	-40(10%)	-94 (28 %)	-134(36%)
MRI-CGCM3	-8 (1 %)	+63(10%)	+3 (1 %)	+77(12%)	0 (0 %)	+60(29%)	+36 (13 %)	+121(50%)	-8 (2 %)	+3(1%)	-32 (9 %)	-44(11%)
**5GCM-NSBL**	***-10 (2 %)***	***+41(7%)***	***+30 (5 %)***	***+75(12%)***	***+3 (3 %)***	***+50(37%)***	***+60 (30 %)***	***+122(73%)***	***-14 (3 %)***	***-9(2%)***	***-30 (7%)***	***-47(10%)***
**11GCM-NSBL**	***-3 (<1 %)***	***+20(4%)***	***-3 (1 %)***	***+18(3%)***	***+9 (6 %)***	***+50(29%)***	***+59 (26 %)***	***+114(57%)***	***-12 (3 %)***	***-36(8%)***	***-63 (16 %)***	***-100(24%)***
**16GCM-NSBL**	***-5 (1 %)***	***+26(4%)***	***+7 (1 %)***	***+36(6%)***	***+5 (4 %)***	***+50(31%)***	***+60 (27 %)***	***+117(61%)***	***-12 (3 %)***	***-27(6%)***	***-52 (12 %)***	***-84(19%)***
Livneh-2015	+31 (5 %)	###	###	###	-16 (14 %)	###	###	###	+47 (9 %)	###	###	###

<u>Annual precipitation over the West Coast</u>

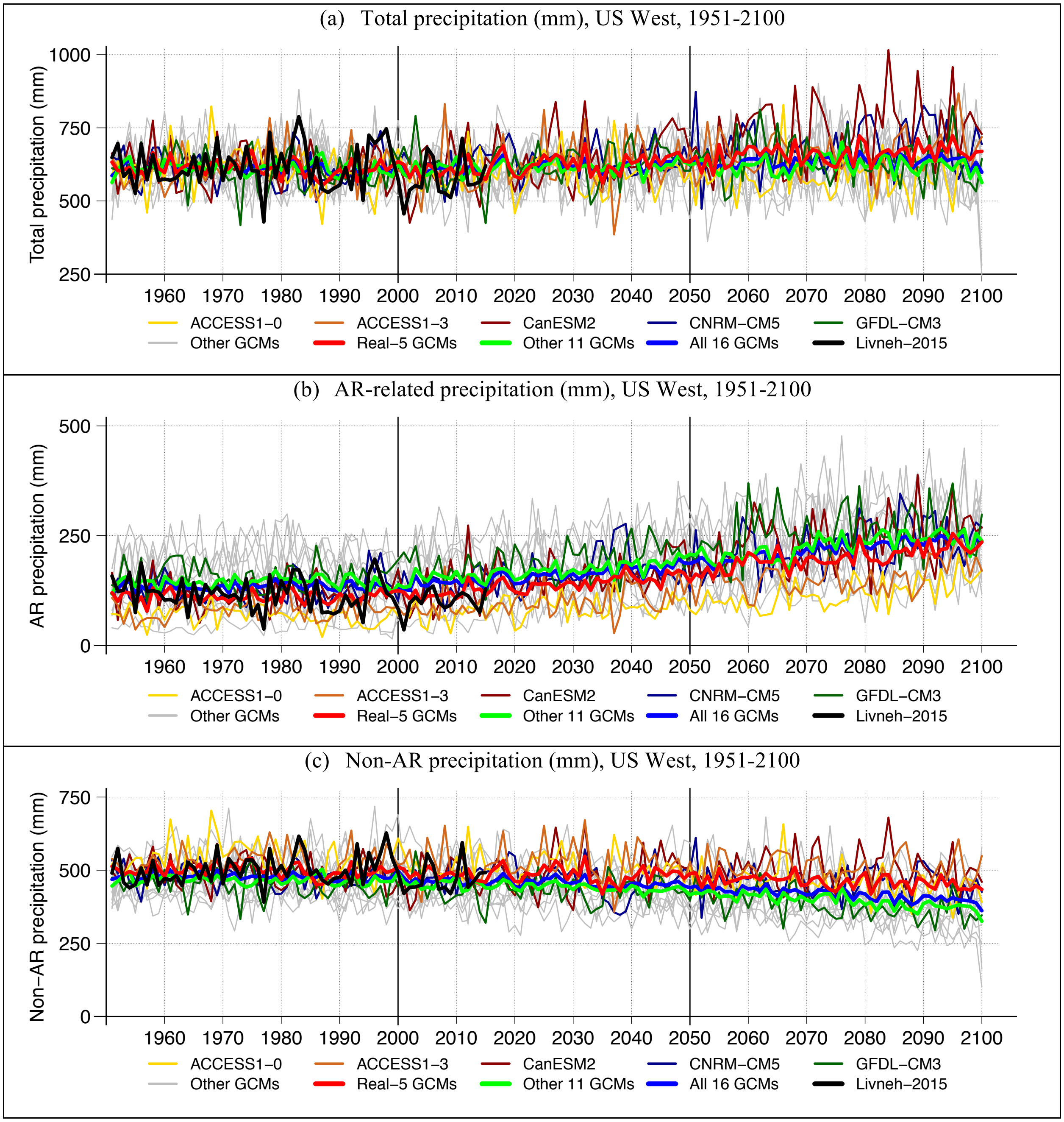

Figure S8. Annual total (a), AR-related (b) and non-AR related (c) LOCA-downscaled precipitation spatially averaged over US West during historical (1951-2005, left) and projected (2007-2100, right) time periods. Results from the Real-5 GCMs are plotted in thin colored lines, while the Other GCMs are outlined in gray. Thick curves represent the ensemble averages of the Real-5 GCMs (red), the Other 11 GCMs (green) and the full ensemble of 16 GCMs (blue). Thick black curve delineates the annual total (a), AR-related (b) and non-AR (c) precipitation, which is based on Livneh precipitation data.
